# Neoadjuvant therapy with cisplatin in BRCA1-positive breast cancer patients

**DOI:** 10.1186/1897-4287-9-S2-A4

**Published:** 2011-06-01

**Authors:** T Byrski, J Gronwald, T Huzarski, RA Dent, D Zuziak, R Wiśniowski, E Marczyk, P Blecharz, O Szurek, C Cybulski, T Dębniak, B Górski, J Lubiński, S Narod

**Affiliations:** 1 International Hereditary Cancer Center, Pomeranian Medical University, Szczecin, Poland; 2 Sunnybrook Odette Cancer Centre, Toronto, ON, Canada; 3 Regional Oncology Center, Bielsko-Biala, Poland; 4 Centre of Oncology Maria Sklodowska-Curie, Memorial Institute Krakow Branch, Poland; 5 Women’s College Research Institute, University of Toronto and Women’s College Hospital, Toronto, Canada

## Background

Neoadjuvant chemotherapy is administered to control disease, make surgical resection possible and increase the possibility of breast tissue conservation. A further advantage of neoadjuvant therapy is that it helps to assess chemo-sensitivity to a particular agent. Induction of a pathological complete response (pCR) is one of the primary goals of neoadjuvant therapy in order to achieve a better disease-free and overall survival. Experimental data suggest that BRCA1 related breast cancer may have increased sensitivity to platinum-based chemotherapy, but clinical data are limited. The aim of this study was to evaluate the frequency of complete pathologic response after neo-adjuvant treatment with cisplatin chemotherapy in women with breast cancer and a BRCA1 mutation.

## Methods

Twenty five women with breast cancer and a BRCA1 mutation with stage I, II, and III breast cancer between December 2006 and July 2009 were entered into this study. Patients were treated with cisplatin 75 mg/m^2^ intravenously every three weeks for four cycles. After chemotherapy, patients underwent surgery and were assessed for pathologic response in both the breast and axillary lymph nodes. Complete pathologic response was defined as no residual invasive disease in both the breast and axilla, however ductal carcinoma in situ was allowed.

## Results

Thirty eight patients were enrolled in the study. Twenty three patients had tumors of greater than two centimeters (60%) and eleven patients had positive lymph nodes at diagnosis (29%). Thirty five patients completed four cycles of cisplatin (92%) and three patients completed two cycles (8%). Clinical complete response was observed in twenty eight patients (74%). Pathologic complete response was observed in twenty three (60,5%).

## Conclusions

Platinum-based chemotherapy is effective in a high proportion of patients with BRCA1-associated breast cancers. Clinical trials are warranted to determine the optimum treatment for this subgroup of breast cancer patients.

